# Reconstructing dinosaur locomotion

**DOI:** 10.1098/rsbl.2024.0441

**Published:** 2025-01-15

**Authors:** Peter L. Falkingham

**Affiliations:** ^1^ School of Biological and Environmental Sciences, Liverpool John Moores University, Liverpool, UK

**Keywords:** dinosaurs, locomotion, biomechanics, modelling, ichnology

## Abstract

Dinosaur locomotor biomechanics are of major interest. Locomotion of an animal affects many, if not most, aspects of life reconstruction, including behaviour, performance, ecology and appearance. Yet locomotion is one aspect of non-avian dinosaurs that we cannot directly observe. To shed light on how dinosaurs moved, we must draw from multiple sources of evidence. Extant taxa provide the basic principles of locomotion, bracket soft-tissue reconstructions and provide validation data for methods and hypotheses applied to dinosaurs. The skeletal evidence itself can be used directly to reconstruct posture, range of motion and mass (segment and whole-body). Building on skeletal reconstructions, musculoskeletal models inform muscle function and form the basis of simulations to test hypotheses of locomotor performance. Finally, fossilized footprints are our only direct record of motion and can provide important snapshots of extinct animals, shedding light on speed, gait and posture. Building confident reconstructions of dinosaur locomotion requires evidence from all four sources of information. This review explores recent work in these areas, with a methodological focus.

## 1. Introduction

The discovery of the first dinosaurs immediately raised questions about how they appeared in life. Fundamental to any such life reconstruction is locomotion; for a terrestrial vertebrate to eat, reproduce, evade predators or play, it needs to be able to move from one point to another. Not only is an understanding of locomotor biology necessary for us to visualize these animals in life, but it is also fundamental to research into other aspects of dinosaur palaeobiology. Questions around predator–prey relationships, metabolism or evolutionary pressures are informed or even dictated by the constraints imposed by locomotor capability.

For nearly three quarters of the time since Buckland named the first dinosaur, dinosaurs were seen as slow, lumbering, cold-blooded animals. The first dinosaur renaissance was triggered by the description of *Deinonychus* [1], helped in large part by Bakker’s illustration in that work of the animal mid-sprint. Changing the prevailing view of how these animals moved, fundamentally changed our views of almost everything else about them.

Dinosaurs present an interesting group for studies of locomotor evolution and mechanics. The group evolved large sizes way beyond what we see in extant terrestrial taxa, and they did so multiple times. The group also inherited bipedalism from their dinosauromorph ancestors and exceeded the size of modern bipeds by an order of magnitude. Some of these bipedal lineages returned to quadrupedality, a rare evolutionary reversion [2,3].

Locomotion is such an important and broad-reaching area of dinosaur palaeontology, that there have of course been prior review articles [4–11]. As such, and given space limitations here, I do not aim to be exhaustive in reviewing all dinosaur locomotion research that has occurred since 1824. This review will focus on recent work, naturally with reference to earlier key papers. I shall generally limit myself to those works whose findings concern non-avian dinosaurs, rather than attempting to also review the vast literature on avian locomotion, and similarly this review will predominantly focus on terrestrial locomotion, with only small consideration of the evolution of flight on the dinosaur to bird lineage.

**Figure 1.**
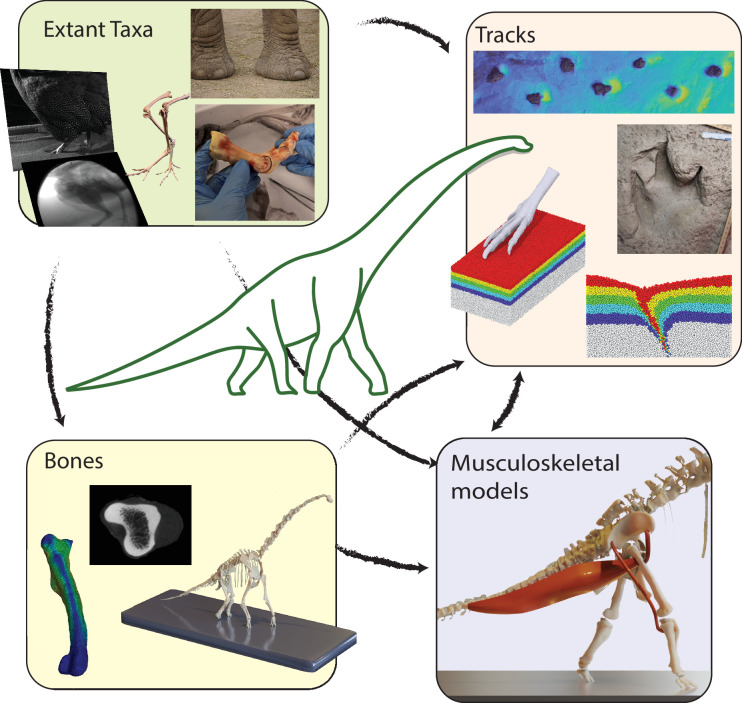
A summary of sources of information about dinosaur locomotion. Extant taxa, either through *in vivo* or *ex vivo* studies, offer the fundamental mechanics of motion, guiding principles and provide validation cases for methods applied to extinct taxa. The skeletons of dinosaurs provide gross information about mass and morphology, as well as clues to loading regimes and posture through bone shape and microstructure. Tracks record direct evidence of gait, speed and distal limb motions, and via simulations can test hypotheses of motion. Musculoskeletal models are constructed from skeletal and extant evidence and can inform us of kinematics and performance metrics of locomotion. Musculoskeletal modelling and tracks can provide useful constraints or hypotheses for each other.

Information about dinosaur locomotion comes from four main sources: extant animals, the fossilized bones themselves, reconstructed soft tissues (inferred from both bones and extant taxa) and fossilized trackways (figure 1). This review shall look at recent work in each of these areas.

## 2. Extant taxa

The methods covered in the following sections all rely on extant taxa for validation, whether that is testing range of motion (ROM) methods on emu feet before applying them to theropods [12], using musculoskeletal models of humans and birds to validate simulations applied to dinosaurs [13] or observing bird track formation to understand how a dinosaur foot likely moved [14]. Indeed, methods for studying dinosaur locomotion should not be used without first being validated against living animals, where accuracy, precision and confidence of the techniques can be established. At the same time, dinosaurs allow us to refine, test and expand on our extant-based ideas. Models derived solely from living animals, for instance, may have flaws or nuances only uncovered when applied to large dinosaurs which extend the mass and size axes well beyond modern taxa [15].

Our fundamental principles of animal locomotion [16,17] are necessarily derived from living animals. It is from living animals that we can draw comparisons, that we can infer soft-tissue morphology and ultimately constrain reconstructions in extinct taxa. Observation of living animals provides a means to discover relationships between form and function that may apply generally, within a clade or morphotype, or throughout a single organism’s lifetime. Because use of extant taxa tends to underpin all of our research avenues into dinosaur locomotion, I will draw attention to the topic throughout subsequent sections. However, it is worth explicitly pointing out some studies that have focused on how living animals move in order to shed light on dinosaur locomotion.

One of the simplest and most commonly encountered metrics of locomotion is speed. Where locomotion as a whole can be a complex system of kinetics and kinematics, speed is a single value that is immediately relatable by everyone; ‘how fast could X dinosaur run’ is one of the most common questions asked by the general public. We shall address how research in dinosaur locomotion has occasionally shed light on this question, but first we must understand the relationship between speed and other factors such as size, locomotor mode and limb length in living animals. This is not as trivial as it may first appear—getting animals to move at their maximum speed in the laboratory or field is notoriously difficult. Alexander reported speeds for numerous fast land animals, encouraging them to run by chasing them in a vehicle from which video was recorded [18], and these values remained the ‘gold standard’ for many years.

The locomotion of large animals is inherently interesting—already high forces resulting from mass and gravity are multiplied during locomotion. This means that limb bones and their associated muscles, ligaments and tendons need to produce, transmit and withstand incredibly large forces [19]. Locomotor studies of large modern animals such as giraffes [20], hippos [21], rhinos [22] and elephants [19,23–25], all serve to shed light on how large animals move and deal with such forces, e.g. through more robust and columnar limb bones.

Unlike many other aspects of animal biology that tend to scale with size, speed has been observed in extant taxa to peak at an ‘intermediate’ size (approx. the size of a cheetah), with smaller and larger taxa attaining lower maximum speeds [26], scaling non-monotonously [27]. Work capacity of muscles constrains maximum speeds of large animals, while physical limits on instantaneous power production of muscles limits top speeds of smaller animals [27,28]. These studies [26–29], while focused on drawing out relationships and mechanisms in extant taxa, all naturally extended their discussions to what this would mean for the largest dinosaurs. Decreasing maximum speed with large size would mean many dinosaurs would be relatively slow (or at least, incapable of high speeds). These studies highlight how study of extant taxa directly informs dinosaur locomotion.

Bipedal locomotion receives much attention both among biologists and palaeontologists, perhaps because it is ‘closer to home’ (in that humans are bipeds), as well as being unusual; only humans and birds are obligate walking/running bipeds today. Dinosaurs represent by far the largest bipeds ever to have existed, potentially encroaching on biomechanical limits [30–33]. Of extant groups, while humans are quite morphologically constrained, birds span several orders of magnitude in size and vary in form. This makes them an excellent group with which to study how and why bipedal locomotion varies [34,35]. By observing living taxa, Bishop *et al.* [34] were able to make inferences about dinosaurs, namely that small and large theropods moving at dynamically similar speeds, would in fact be moving very differently, with smaller animals more crouched.

If any form of locomotion receives as much attention as bipedalism, it is flight. With the flood of feathered dinosaur discoveries over the past three decades [36], understanding how flight is linked to size, age and morphology has been a major area of study, particularly in how it relates to the origins of flight. Dial observed wing-assisted incline running (WAIR; [37]), in which birds use the wings to aid ascent of steep, vertical and even overhanging surfaces. Dial noted that this effect was present even in juveniles without fully formed feathers, and subsequent studies [38–40] have further explored this within ontogenetic sequences of living birds, noting that the ontogenetic trajectories in wing size and feather structure bear many similarities with evolutionary trajectories observed in fossil theropods [38]. It is highly likely that WAIR represents a means for even the most incipient of wings and feathers to provide a benefit on which natural selection can act, offering an explanation for the origin of feathered flight alternative to the more traditional trees-down hypothesis.

Artificially manipulating extant taxa can shed light on how organisms respond to constraints that may have naturally affected dinosaurs. For instance, to study the effects of the large muscular tail on posture and locomotion, two studies [41,42] suspended weights posterior to the hip in chickens, moving the centre of mass back, to test the hypothesis that a large muscular tail in dinosaurs necessitated a more vertical limb posture. The first study [41] observed that the femur actually became more horizontal (though still within the range of other bird taxa) with the addition of posterior weights, counter to the original hypothesis. However the second later study [42] raised chickens from hatching to maturity with posterior weights attached, and in larger enclosures where they could exercise. This second study [42] did observe a more vertically oriented femur as predicted by the original hypothesis.

## 3. Bones

The fossilized bones of dinosaurs are our primary source of information about almost all aspects of their palaeobiology, and it is only rarely that skeletons do not take centre stage in any museum exhibit. Skeletal elements can be used to infer size and mass, limb lengths and ROM at a given joint, all of which can constrain possible locomotor performance or provide inputs to more advanced modelling (see next section).

### (a) Scaling, size and mass

Acknowledging relatively small uncertainties due to cartilage and other soft tissues, skeletons can directly tell us how long and tall an animal was, and how long its limbs were, providing at the very least a point of comparison with extant taxa. A less directly measurable, but equally important metric is mass, which fundamentally affects how an animal can move [27,28,43,44]. Specifically mass dictates how much force muscles need to generate. In addition to absolute mass, centre of mass and moments of inertia have major effects on posture and manoeuvrability [4,45]. Mass is generally determined in one of the two ways, either through scaling equations or through three-dimensional modelling [46].

Scaling equations rely on relationships between size of specific bones (usually the humerus or femur or some combination of limb bones), and overall body mass, and are derived from living taxa [47–50]. This has clear advantages over the more complex process of three-dimensional modelling, in that mass can be calculated for many individuals or species with only a few measurements, enabling rapid and extensive data collection, which can be necessary for broad evolutionary studies. They are also applicable to relatively incomplete skeletons, requiring only a single bone, or even potentially a partial bone, and do not rely on soft-tissue reconstruction. However, the scaling relationships are derived from extant taxa, all of which are smaller than the largest dinosaurs, and often morphologically very different. It is, therefore, difficult to test if scaling equations are usefully accurate outside of the dataset from which they are determined.

The alternative is to reconstruct the full form of the dinosaur to calculate mass. Alexander immersed scaled dinosaur models in water to calculate volume and multiplied by an assumed density of 1000 kg m^−3^ [5]. Today, the process predominantly occurs virtually via digital three-dimensional modelling—reconstructing a soft-tissue outline around the skeleton [46,51,52]. This method naturally requires a relatively complete specimen and is inherently more involved to calculate than measuring a single bone, though modern digitization techniques [51,53,54], and hulling methods [55,56] have greatly simplified this process. Volumetric models can be wholly multiplied by assumed densities ([55]; often lower than 1000 kg m^−3^ to account for the pneumaticity present in many dinosaurs), or the models may be constructed with distinct approximate volumes for bone, muscle and air sacs+lungs, and each multiplied by a particular density [57,58].

Both methods, scaling and volumetric modelling, continue to be used, offering differing advantages. Scaling equations have been described as having accuracy, and volumetric models as providing precision [50], though there remain some cases of significant disagreement between the methods (e.g. [59,60] where the mass of *Dreadnoughtus* was calculated as approx. 60 t using scaling equations, versus approx. 27 t with volumetric modelling) inviting further work refining these methods [50].

### (b) Range of motion

The ROM of any given joint is integral to how the limb and body move, providing hard and soft constraints that limit the possible postures and movements. Joint mobility has been described as a bridge between form and function [61]. At its most basic level, research into dinosaur ROM involves taking the bones, either real, cast or digital, and manipulating them to find hard stops that limit further movement at a joint [62–66].

However, we know from numerous *in vivo* and *ex vivo* studies [12,67–69], especially those using X-ray Reconstruction of Moving Morphology [70–74], that joint ROM is highly dependent on soft tissues, which can not only alter the shape of the joint relative to the bones, but can actively deform, enabling translation of the axis of rotation. Combining progressive dissections, in which soft tissues are systematically removed [75,76] can provide more rigorous investigations into dinosaur ROM [12,77,78], but even here, the values recovered can be subjective and dependent on the rotation axes in which the observer moves the bones. Because the seemingly simple task of determining ROM has proven to be so complex, recent work has advanced methods to measure ROM in a standardized manner [74,79–81] which will in the future facilitate more comparative studies.

Bones and joint ROM are enough to start to constrain limb poses and body postures, and infer facets of locomotion, without knowledge of soft-tissues. Gatesy *et al.* [82] used constraint-based exclusion of limb poses to reconstruct *Tyrannosaurus* limb posture at mid stance, validating their method against extant ratites. Starting with all possible limb poses, the ‘configuration space’ was whittled down by removing least probable poses. Joint ROMs were the first and most significant constraint. Other kinetic constraints of increasing uncertainty (e.g. resultant moment signs and magnitudes) further reduced possible poses until only a narrow range of limb configurations remained. The reconstructed limb poses indicated only slow running at best for *Tyrannosaurus rex*.

### (c) Bone structure

Both the internal and external structures of bones provide crucial insights into dinosaur locomotion. Gross morphological features, particularly the scaling of proportions with body size, offer clues about locomotor capabilities across dinosaur taxa. Limb proportions and overall body plan change systematically with increasing body mass, reflecting biomechanical constraints and adaptations [44,83]. Allometric scaling, where different body parts grow at different rates in relation to body size, plays a critical role in these changes. For instance, larger theropods tend to have relatively short and more robust limb bones compared with their smaller counterparts, a trend also observed in modern mammals [84]. This allometric scaling has significant implications for speed, agility and overall locomotor performance [43,85]; shorter limbs reduce relative stride length, reducing speed; robust limbs resist bending and compressive forces but increase mass and cost of transport.

At a smaller scale, the microstructure of dinosaur bones provides a direct record of the loads experienced during life, offering invaluable data on posture and movement patterns. Cortical bone cross-sectional geometry reflects the dominant loading regimes experienced by the limb bones [86,87]. For example, the thick-walled, elliptical cross-sections often observed in theropod femora suggest adaptation to both bending and torsional loads, consistent with an upright, parasagittal gait [83]. Cubo *et al*. [88] provided evidence of biomechanically adaptive bone modelling, changes in structure due to stress, in the hadrosaur *Maiasaura*, demonstrating how bone microstructure can change during growth in response to mechanical loads. That these changes occur within a single taxon is informative as to ontogenetic changes in locomotor capabilities throughout ontogeny in at least some large dinosaurs.

While bone morphology offers information about posture and movement, and skeletons of dinosaurs are a natural starting point for studying their locomotion, many uncertainties remain, particularly regarding the drivers of motion, muscles. To further refine our understanding of dinosaur locomotion, we need to move ‘beyond the bones’ [9].

## 4. Muscles, soft-tissues and computers

### (a) Reconstructing muscles

While it is the osteology that provides the basis of our understanding of dinosaur locomotion, the skeletons cannot move on their own (museum visits might be far more exciting were that the case). Skeletons are driven by muscles, which, for all intents and purposes here, do not fossilize, making biomechanical reconstructions difficult [89]. Muscle is highly conserved across Tetrapoda [17,90], so we can with some confidence reconstruct the properties of dinosaur muscle. Where the uncertainties lie is in which muscles a given dinosaur had, the path said muscles took, and the size of those muscles. Ascertaining if a given muscle crossed a joint, and how large it was, can give an indication of the power the animal might exert at said joint.

Osteological evidence in the form of muscle scars, rough areas where tendons attached muscles to bone, can provide primary information about the presence of muscles [91]. However, not all muscles leave evidence on the bones, and not all skeletons (in fact, very few) are complete, so the extant phylogenetic bracket [92] is employed to provide levels of confidence as to whether a dinosaur possessed any given muscle.

When muscle scars are present, they can describe the origin and insertion points of a muscle, which in turn determines which joint or joints would be acted upon. Myological reconstructions of dinosaurs have been carried out increasingly often, and across all major groups [7,31,93–103]. The rapidly growing literature on muscle reconstructions in dinosaurs has in large part been facilitated by the increasing availability of digitization methods, which facilitate observation of muscle scars and placing muscles accurately in three-dimensional space without needing to manhandle large fragile specimens.

By reconstructing muscle locations, moment arms (or lever arms) can be calculated; the distance between the joint centre and the line of action of the muscle [31,97–99,103]. Larger moment arms create greater moments about a joint, reducing the absolute force required to move the joint but require more muscle length change and faster velocities than smaller moment arms, for the same rotations. Hutchinson & Garcia’s seminal work [32] on *Tyrannosaurus* used reconstructed moment arms to estimate the minimum mass of muscle required to counteract ground reaction forces experienced during running and showed that given said moment arms, the mass of muscle required was unreasonably large; subsequently they determined *Tyrannosaurus* and other large theropods were not capable of fast running. The speed of *T. rex* has remained a topic of interest for musculoskeletal studies [31,104–106], mostly supportive of a slower maximum speed.

Reconstructing muscle locations, sizes and moment arms can be deeply informative, and recent work has made efforts to document and standardize the process of building these musculoskeletal models [107] and constraining their inputs around soft-tissue parameters [108]. But muscle force production is an area of uncertainty [109]. Ultimately, muscle force is determined by physiological cross-sectional area, which is defined as muscle mass divided by fibre length. Fibre length can be estimated (with caveats around pennation) from the muscle length, as defined by origin and insertion. But muscle mass, or volume, is much harder to constrain from osteological information. Some muscles, like the caudofemoralis which might be constrained by caudal vertebral osteology (e.g. [110]) can be estimated with some confidence, but for the most part, muscle volume reconstructions are subjective and require sensitivity analyses [111,112] to limit possible reconstructions and place minima and maxima on possible force production.

### (b) Dynamic musculoskeletal modelling

If we have a digital model of a dinosaur, which includes the bones, (ranges for) the masses of body segments, as well as the muscle positions and volumes, we have the building blocks to make a full dynamic model. Sellers *et al.* [13] were the first to build such dynamic models for dinosaurs, utilizing evolutionary algorithms to find optimal sequences and magnitudes with which to activate the included muscle groups. Such a method requires significant computational resources, and indeed Hutchinson & Gatesy’s review the prior year stated ‘rigorous dynamic simulation of a moving dinosaur, one encompassing all motions and forces, cannot yet plausibly be done’ [9]. Sellers *et al.* were able to generate gaits for several non-avian theropods plus emu, ostrich and humans as validation taxa. The simulated extant taxa achieved maximum running speeds close to what is observed *in vivo*, and predicted unexceptional speeds for the non-avian theropods (8–10 m s^−1^), with the larger taxa, *Allosaurus* and *Tyrannosaurus* moving slower than the smaller taxa *Velociraptor* and *Dilophosaurus*, broadly in line with the trends of speed and size observed in extant taxa, outlined earlier [26].

Subsequent studies have also incorporated dynamic modelling to investigate bipedal locomotion in theropods [106,112,113], possible gaits and facultative/obligate bipedalism in ornithopods [114] and locomotion in the very largest dinosaurs, sauropods [15]. These studies shed light not only on maximum speeds [13], but on most efficient gaits [15], the contribution of body parts such as the tail to overall movement of the body [105,113] and the further constraints of stresses and strains within the bones [106]. A strength of musculoskeletal modelling is that the simulations are not only based on anatomical evidence but are fundamentally based on physics, enabling us to find hard limits and constraints on what is biologically possible, even for taxa for which there are no modern analogues.

## 5. Dinosaur tracks and trackways

Bones are a direct record of the animal’s morphology, but it is fossil footprints, or tracks, which are direct records of motion. The three-dimensional morphology of a given track is determined by the anatomy of the foot and distal limb, the consistency and response of the substrate and the kinetics and kinematics of the foot that made it [115]. Trackway parameters such as stride length, pace angulation and foot rotation, are all 1 : 1 records of foot placement, and therefore represent posture, speed and behaviour. However, this information is tempered by two limitations: first, that the identification of a trackmaker can be notoriously difficult, certainly to species level, but even to much higher taxonomic levels. Second, that the shape of a fossil track is a result not only of the formational process itself but also subsequent preservation and exposure processes [116,117].

The scientific literature on dinosaur tracks extends almost as far back as Buckland’s naming of *Megalosaurus,* with Hitchcock’s reports on the tracks of the Connecticut valley [118–120]. Indeed, even in Hitchcock’s earliest publications on the subject, he was able to use tracks to make statements about the trackmaker’s locomotion: *‘I suspect from the numerous examples which I have seen of tracks at the distance of four feet, that this was the ordinary step of the bird when walking; while it was able to lengthen it to six feet, when moving rapidly*’.

Tracks and trackways tend to be used to infer locomotion at two scales. Individual tracks are used to infer specific movements of the autopodia. Trackways and tracksites tend to be used as evidence of whole-animal movements of individuals and groups.

### (a) Trackways, gaits and speed

One of the most ubiquitous applications of trackways to locomotor research is Alexander’s equation for calculating speed from stride lengths [121]. Noting that stride length typically increases as an animal moves faster, Alexander derived a relationship with which to predict speed from trackways. Integral to this equation is hip height, which while not recorded directly in trackways, was estimated by Alexander as four times foot length. Subsequent authors made alterations to Alexander’s equation to account for faster dinosaurs [18,122] or to more accurately calculate hip height for specific clades [123]. Despite being heavily cited over the past approximately 50 years, it is worth pointing out that Alexander himself cautioned against relying on numbers output by the equation, noting in 2006 [4] that ‘*The method cannot claim to be accurate. We cannot be certain that the relationship ... was the same for dinosaurs as for mammals… The method depends on doubtful estimates of leg length… Though it cannot predict precise speeds, the method is informative; there seems to be no likelihood of confusing a stroll with a sprint’.* Multiple authors since have commented similarly [124–126], yet the method remains very widely used.

Determining what gait, or pattern of footfalls, quadrupedal dinosaurs used is difficult. In quadrupeds, front and hind feet can vary timing significantly, yet still produce the same footfall pattern on the ground, resulting in the same trackway parameters. Distinguishing a trot from a pace, for instance, has until recently not been possible from trackways. However, recent papers [127,128] have used variability in stride lengths along a trackway to constrain gleno-acetabular distance, and subsequently to limit possible limb phases, and therefore gait, of the trackmaker. Both papers found sauropod trackways indicating a diagonal couplets gait (or a limb phase around 0.3). Trackways can also give insight into manoeuvrability of dinosaurs, with several reports of turning sauropods [129–133] indicating particularly tight turns while maintaining footfall patterns.

Bipedal gaits are simpler in that there are fewer possible gaits (walking, running and grounded running), though how bipeds transition between these gaits is of interest. Work by Bishop *et al.* [134] used trackway width, in conjunction with stride (speed) to determine that non-avian theropods likely had a continuous locomotor repertoire, moving gradually from walking to running, as in modern birds and unlike humans who switch abruptly from a walk to a run.

Trackways also provide insight into posture. Sauropod trackways have been divided into ‘narrow’ and ‘wide’ gauge based on the internal distance between left and right tracks, with wider gauge trackways having been attributed to titanosaurs [135]. However, while many sauropod trackways may be distinctly ‘narrow’ or wide’ gauged [132,135–138], there are reports of trackways which change in width, or ‘gauge’ along their length [139].

In cases where osteology or musculoskeletal simulations may not decisively indicate whether an animal was bipedal or quadrupedal (e.g. hadrosaurs), throughout part or all of its life, tracks can provide definitive evidence [140]. Although many trackways may not be definitive due to overprinting (the pes stepping where the manus was, obliterating the manus track), presence of manus impressions within a trackway offers indisputable evidence of at least facultative quadrupedality [141].

Because the hind foot naturally comes *after* the manus, overprinting by the pes is a common and expected phenomena, resulting in pes-only or pes-dominated trackways [142,143]. However, the reverse situation can also be found—manus only or manus-dominated trackways, as seen in numerous reports from sauropod tracksites [144–147]. These cases present a bigger challenge to interpret—how can the pes impression be missing, when the manus is clearly indented? Early interpretations invoked swimming ([148–150], or technically ‘punting’ because feet had to contact the bottom to make tracks [151]). However, modelling footprint formation while taking into account centre of mass position and differential pressures under the manus and pes have suggested a terrestrial origin for many of these trackways, whereby the higher pressures resulting from the smaller manus likely overcame the sediment strength, leaving tracks only under the front feet [143,152].

Theropods also have a record of enigmatic or unusual traces. Descriptions of ‘swim’ tracks and trackways are common in the literature, often expressed as elongated scratch-like marks [153–158] caused by the tips of the claws just reaching the surface of the sediment. Elongate scratch-like impressions have even been attributed to swimming stegosaurs [159]. However, similar track morphologies of elongate toe marks have been noted toward the bottom of volumetric tracks made in soft mud [116,160], where the distinction between walking and swimming becomes much less apparent.

### (b) Individual tracks

Singular tracks, preserved isolated or within a trackway, can be rich sources of information about specific movements of the distal limb [160–164]. Because the three-dimensional morphology of the track (at the surface, and throughout the track volume [165–167]) is the result of foot motion, it is possible to ‘reverse engineer’ the track to trace the path the foot must have taken [14,168,169]. Recent work has used particle simulation to model the foot–sediment interaction and directly test whether the reconstructed motions are possible, and whether they produce virtual tracks that match the fossils, essentially testing ‘hypotheses of motion’ [160]. Most dinosaur-track foot kinematics reconstructions have used theropod tracks, and recovered sweeping motions with generally low entry and exit angles, often bearing similarities with extant birds [14,162]. Deep sauropod manus tracks have tended to indicate a more vertical ‘stamping’ motion of the foot [170,171], though many sauropod pes impressions do display a forward exiting motion of the foot, resulting in lifting of the sediment at the anterior of the track.

### (c) Limitations

Using tracks to reconstruct foot motions suffers from an inconvenient fact: the deeper the foot sinks, the more motion is recorded and can subsequently be recovered; however, the deeper the animal sinks the less likely that the recorded motion is ‘stereotypical’ of the animal walking on firmer surfaces. Drawing comparisons between dinosaur locomotion and that of modern taxa, particularly birds, therefore may necessitate having the animals move over similarly compliant substrates [14,116,162,168,172].

Another drawback to fossil footprints is that they will rarely record maximal performance. The prior sections on osteological and myological locomotor studies tend to focus on how limbs, joints, and muscles can maximise force, or minimize stress, and subsequently tend to find extremes of plausible locomotor mechanics. But animals rarely move with maximum performance, and when they do it is even more rarely that it might be on a compliant substrate capable of deforming into tracks, deep or otherwise. Trackways are, therefore, unlikely to answer questions about how fast a trackmaker could run, or even if it could run at all. This is borne out by most trackways [121,173–175], with few exceptions [174,176], indicating unexceptional ‘normal’ walking speeds for the trackmakers.

## 6. Summary and future directions

I have outlined above our major sources of information about dinosaur locomotion:

— extant taxa: from which principles of animal locomotion can be derived, as well as scaling relationships, and links between form and function;
— bones: our basis of life reconstructions, and from which key locomotor parameters such as mass, limb lengths, joint mobility, and muscle positions and actions can be determined;
— soft-tissues: the muscles, tendons and ligaments that would have generated and transmitted forces to the bones, driving locomotion;
— tracks and trackways: direct evidence of fossilized motion, recording gait, behaviours and speeds.

Each source of information brings unique insights. Each also has weaknesses; extant taxa do not occupy the full size or morphospace that dinosaurs did, and we cannot say with certainty that extrapolations drawn only from living taxa are necessarily accurate. Bones and reconstructed muscles both constrain possible locomotor capabilities through limits on ROM and maximal force generation, respectively. But these reconstructions often leave wide ranges of plausible outcomes and carry numerous assumptions arising from the vagaries of reconstructing missing soft-tissues in animals with poor extant functional analogues. Musculoskeletal research also often focuses on extremes—maximal speed, maximal mobility, etc., which do not necessarily inform us about stereotypical locomotion. Conversely, being direct records of motion, tracks can offer locomotor insights and a level of confidence that is not always present from body fossils alone, but the certainty that a pes fell exactly in this position, and a manus in this one, is tempered by difficulties in assigning which dinosaur made the tracks in the first place, and by rarely recording anything approaching maximal performance.

The key to advancing our understanding of dinosaur locomotion lies in closer integration of all sources of information such that validation, theory, simulation and primary data come together. While there are clear connections between extant taxa and dinosaur osteology and myology, there is a potential for further refinement by enhancing the complexity of musculoskeletal models (aided by ongoing improvements in computational capability) and increasing the validation of these simulations. Integrating constraints from bone mechanics and coupling these with validation against extant taxa, we can achieve unprecedented confidence in our reconstructions of dinosaur locomotion.

Further coupling of the ichnological record with more traditional biomechanics studies presents may be the most significant opportunity to advance our understanding of dinosaur locomotion. This approach bridges the fossil record of motion with the fossil record of form. Dinosaur tracks offer a critical test for hypotheses of motion derived only from anatomy and physics. For instance, comparing footfall patterns from forward dynamics simulations with those observed in the track record. Recovery of specific foot motions from deep penetrative fossil tracks may act as distal constraints for limb-based musculoskeletal models. These models, given enough computing resource, might incorporate sediment simulations directly creating a realistic feedback loop between muscles and environment and producing directly testable hypotheses.

## Data Availability

This article has no additional data.

## References

[1] Ostrom JH. 1969 Osteology of Deinonychus antirrhopus, an unusual theropod from the Lower Cretaceous of Montana. Bull. Peabody Mus. Nat. Hist. **30**.

[2] Maidment SCR, Linton DH, Upchurch P, Barrett PM. 2012 Limb-bone scaling indicates diverse stance and gait in quadrupedal ornithischian dinosaurs. PLoS One **7**, e36904. (10.1371/journal.pone.0036904) 22666333 PMC3358279

[3] Maidment SCR, Henderson DM, Barrett PM. 2014 What drove reversions to quadrupedality in ornithischian dinosaurs? Testing hypotheses using centre of mass modelling. Naturwissenschaften **101**, 989–1001. (10.1007/s00114-014-1239-2) 25228349

[4] Alexander RM. 2006 Dinosaur biomechanics. Proc. R. Soc. B **273**, 1849–1855. (10.1098/rspb.2006.3532) PMC1634776 16822743

[5] Alexander RMcN. 1985 Mechanics of posture and gait of some large dinosaurs. Zool. J. Linn. Soc. **83**, 1–25. (10.1111/j.1096-3642.1985.tb00871.x)

[6] Farlow JO, Gatesy SM, Holtz Jr TR, Hutchinson JR, Robinson JM. 2000 Theropod locomotion. Am. Zool. **40**, 640–663. (10.1668/0003-1569(2000)040[0640:tl]2.0.co;2)

[7] Gatesy SM. 1995 Functional evolution of the hind limb and tail from basal theropods to birds. In Functional morphology in vertebrate paleontology (ed. J Thomason), pp. 219–234. Cambridge, UK: Cambridge University Press.

[8] Hutchinson JR. 2006 The evolution of locomotion in archosaurs. Cr. Palevol. **5**, 519–530. (10.1016/j.crpv.2005.09.002)

[9] Hutchinson JR, Gatesy SM. 2006 Beyond the bones. Nature **440**, 292–294. (10.1038/440292a) 16541062

[10] Demuth OE, Herbst E, Polet DT, Wiseman ALA, Hutchinson JR. 2023 Modern three-dimensional digital methods for studying locomotor biomechanics in tetrapods. J. Exp. Biol. **226**, b245132. (10.1242/jeb.245132) PMC10042237 36810943

[11] Otero A, Hutchinson JR. 2022 Body size evolution and locomotion in Sauropodomorpha: what the South American record tells us. In South american sauropodomorph dinosaurs: record, diversity and evolution (eds A Otero, JL Carballido, D Pol), pp. 443–472. Cham, Switzerland: Springer. (10.1007/978-3-030-95959-3_12)

[12] White MA, Cook AG, Klinkhamer AJ, Elliott DA. 2016 The pes of Australovenator wintonensis (Theropoda: Megaraptoridae): analysis of the pedal range of motion and biological restoration. PeerJ **4**, e2312. (10.7717/peerj.2312) 27547591 PMC4975041

[13] Sellers WI, Manning PL. 2007 Estimating dinosaur maximum running speeds using evolutionary robotics. Proc. R. Soc. B **274**, 2711–2716. (10.1098/rspb.2007.0846) PMC2279215 17711833

[14] Falkingham PL, Gatesy SM. 2014 The birth of a dinosaur footprint: subsurface 3D motion reconstruction and discrete element simulation reveal track ontogeny. Proc. Natl Acad. Sci. USA **111**, 18279–18284. (10.1073/pnas.1416252111) 25489092 PMC4280635

[15] Sellers WI, Margetts L, Coria RA, Manning PL. 2013 March of the titans: the locomotor capabilities of sauropod dinosaurs. PLoS One **8**, e78733. (10.1371/journal.pone.0078733) 24348896 PMC3864407

[16] Alexander RM. 2003 Principles of animal locomotion. Princeton, NJ: Princeton University Press. (10.1515/9781400849512)

[17] Biewener AA. 2003 Animal locomotion, p. 281. Oxford, UK: Oxford University Press.

[18] Alexander RMcN, Langman VA, Jayes AS. 1977 Fast locomotion of some African ungulates. J. Zool. **183**, 291–300. (10.1111/j.1469-7998.1977.tb04188.x)

[19] Hutchinson JR. 2021 The evolutionary biomechanics of locomotor function in giant land animals. J. Exp. Biol. **224**, b217463. (10.1242/jeb.217463) PMC8214834 34100541

[20] Basu C, Wilson AM, Hutchinson JR. 2018 The locomotor kinematics and ground reaction forces of walking giraffes. J. Exp. Biol. b159277. (10.1242/jeb.159277) 30510118

[21] Hutchinson JR, Pringle EV. 2024 Footfall patterns and stride parameters of common hippopotamus (Hippopotamus amphibius) on land. PeerJ **12**, e17675. (10.7717/peerj.17675) 38974416 PMC11227274

[22] Etienne C, Houssaye A, Fagan MJ, Hutchinson JR. 2024 Estimation of the forces exerted on the limb long bones of a white rhinoceros (Ceratotherium simum) using musculoskeletal modelling and simulation. J. Anat. **245**, 240–257. (10.1111/joa.14041) 38558391 PMC11259748

[23] Hutchinson JR, Schwerda D, Famini DJ, Dale RHI, Fischer MS, Kram R. 2006 The locomotor kinematics of Asian and African elephants: changes with speed and size. J. Exp. Biol. **209**, 3812–3827. (10.1242/jeb.02443) 16985198

[24] Miller CE, Basu C, Fritsch G, Hildebrandt T, Hutchinson JR. 2008 Ontogenetic scaling of foot musculoskeletal anatomy in elephants. J. R. Soc. Interface **5**, 465–475. (10.1098/rsif.2007.1220) 17974531 PMC2607390

[25] Hutchinson JR, Miller C, Fritsch G, Hildebrandt T. 2002 The anatomical foundation for multidisciplinary studies of animal limb function: examples from dinosaur and elephant limb imaging studies. Wildlife Res. 23–38. (10.1007/978-4-431-76933-0_3)

[26] Hirt MR, Jetz W, Rall BC, Brose U. 2017 A general scaling law reveals why the largest animals are not the fastest. Nat. Ecol. Evol. **1**, 1116–1122. (10.1038/s41559-017-0241-4) 29046579

[27] Labonte D, Bishop PJ, Dick TJM, Clemente CJ. 2024 Dynamic similarity and the peculiar allometry of maximum running speed. Nat. Commun. **15**, 2181. (10.1038/s41467-024-46269-w) 38467620 PMC10928110

[28] Usherwood JR, Gladman NW. 2020 Why are the fastest runners of intermediate size? Contrasting scaling of mechanical demands and muscle supply of work and power. Biol. Lett. **16**, 20200579. (10.1098/rsbl.2020.0579) 33023380 PMC7655479

[29] Dick TJM, Clemente CJ. 2017 Where have all the giants gone? How animals deal with the problem of size. PLoS Biol. **15**, e2000473. (10.1371/journal.pbio.2000473) 28076354 PMC5226675

[30] Rhodes MM, Henderson DM, Currie PJ. 2021 Maniraptoran pelvic musculature highlights evolutionary patterns in theropod locomotion on the line to birds. PeerJ **9**, e10855. (10.7717/peerj.10855) 33717681 PMC7937347

[31] Hutchinson JR, Anderson FC, Blemker SS, Delp SL. 2005 Analysis of hindlimb muscle moment arms in Tyrannosaurus rex using a three-dimensional musculoskeletal computer model: implications for stance, gait, and speed. Paleobiology **31**, 676. (10.1666/04044.1)

[32] Hutchinson JR, Garcia M. 2002 Tyrannosaurus was not a fast runner. Nature **415**, 1018–1021. (10.1038/4151018a) 11875567

[33] Persons WS, Currie PJ, Erickson GM. 2019 An older and exceptionally large adult specimen of Tyrannosaurus rex. Anat. Rec. **303**, 656–672. (10.1002/ar.24118) 30897281

[34] Bishop PJ *et al*. 2018 The influence of speed and size on avian terrestrial locomotor biomechanics: predicting locomotion in extinct theropod dinosaurs. PLoS One **13**, e0192172. (10.1371/journal.pone.0192172) 29466362 PMC5821450

[35] Gatesy SM, Biewener AA. 1991 Bipedal locomotion: effects of speed, size and limb posture in birds and humans. J. Zool. **224**, 127–147. (10.1111/j.1469-7998.1991.tb04794.x)

[36] Ksepka DT. 2020 Feathered dinosaurs. Curr. Biol. **30**, R1347–R1353. (10.1016/j.cub.2020.10.007) 33202226

[37] Dial KP. 2003 Wing-assisted incline running and the evolution of flight. Science **299**, 402–404. (10.1126/science.1078237) 12532020

[38] Heers AM, Dial KP, Tobalske BW. 2014 From baby birds to feathered dinosaurs: incipient wings and the evolution of flight. Paleobiology **40**, 459–476. (10.1666/13057)

[39] Dial TR, Heers AM, Tobalske BW. 2012 Ontogeny of aerodynamics in mallard ducks: comparative performance and developmental implications. J. Exp. Biol. **215**, 3693–3702. (10.1242/jeb.062018) 22855612

[40] Heers AM, Tobalske BW, Dial KP. 2011 Ontogeny of lift and drag production in ground birds. J. Exp. Biol. **214**, 717–725. (10.1242/jeb.051177) 21307057 PMC3036546

[41] Carrano MT, Biewener AA. 1999 Experimental alteration of limb posture in the chicken (Gallus gallus) and its bearing on the use of birds as analogs for dinosaur locomotion. J. Morphol. **240**, 237–249. (10.1002/(sici)1097-4687(199906)240:33.3.co;2-e) 10367398

[42] Grossi B, Iriarte-Díaz J, Larach O, Canals M, Vásquez RA. 2014 Walking like dinosaurs: chickens with artificial tails provide clues about non-avian theropod locomotion. PloS One **9**, e88458. (10.1371/journal.pone.0088458) 24505491 PMC3915051

[43] Bishop PJ, Bates KT, Allen VR, Henderson DM, Randau M, Hutchinson JR. 2020 Relationships of mass properties and body proportions to locomotor habit in terrestrial Archosauria. Paleobiology **46**, 550–568. (10.1017/pab.2020.47)

[44] Gatesy SM. 1991 Hind limb scaling in birds and other theropods: implications for terrestrial locomotion. J. Morphol. **209**, 83–96. (10.1002/jmor.1052090107) 29865536

[45] Carrier DR, Walter RM, Lee DV. 2001 Influence of rotational inertia on turning performance of theropod dinosaurs: clues from humans with increased rotational inertia. J. Exp. Biol. **204**, 3917–3926. (10.1242/jeb.204.22.3917) 11807109

[46] Brassey CA. 2016 Body-mass estimation in paleontology: a review of volumetric techniques. Paleontol. Soc. Pap. **22**, 133–156. (10.1017/scs.2017.12)

[47] Campione NE. 2017 Extrapolating body masses in large terrestrial vertebrates. Paleobiology **43**, 693–699. (10.1017/pab.2017.9)

[48] Campione NE, Evans DC, Brown CM, Carrano MT. 2014 Body mass estimation in non‐avian bipeds using a theoretical conversion to quadruped stylopodial proportions. Methods Ecol. Evol. **5**, 913–923. (10.1111/2041-210x.12226)

[49] Campione NE, Evans DC. 2012 A universal scaling relationship between body mass and proximal limb bone dimensions in quadrupedal terrestrial tetrapods. BMC Biol. **10**, 60. (10.1186/1741-7007-10-60) 22781121 PMC3403949

[50] Campione NE, Evans DC. 2020 The accuracy and precision of body mass estimation in non‐avian dinosaurs. Biol. Rev. **95**, 1759–1797. (10.1111/brv.12638) 32869488

[51] Bates KT, Manning PL, Hodgetts D, Sellers WI. 2009 Estimating mass properties of dinosaurs using laser imaging and 3D computer modelling. PloS One **4**, e4532. (10.1371/journal.pone.0004532) 19225569 PMC2639725

[52] Henderson DM. 2006 Burly gaits: centers of mass, stability, and the trackways of sauropod dinosaurs. J. Vertebr. Paleontol. **26**, 907–921. (10.1671/0272-4634(2006)26[907:bgcoms]2.0.co;2)

[53] Falkingham P. 2012 Acquisition of high resolution three-dimensional models using free, open-source, photogrammetric software. Palaeontol. Electron. **15**. (10.26879/264)

[54] Mallison H. 2011 Digitizing methods for paleontology: applications, benefits and limitations. In Computational paleontology (ed. AMT Elewa), pp. 7–43. Heidelberg, Germany: Springer Berlin. (10.1007/978-3-642-16271-8_2)

[55] Sellers WI, Hepworth-Bell J, Falkingham PL, Bates KT, Brassey CA, Egerton VM, Manning PL. 2012 Minimum convex hull mass estimations of complete mounted skeletons. Biol. Lett. **8**, 842–845. (10.1098/rsbl.2012.0263) 22675141 PMC3440966

[56] Bates KT, Falkingham PL, Hodgetts D, Farlow JO, Breithaupt BH, O’Brien M, Matthews N, Sellers WI, Manning PL. 2009 Digital imaging and public engagement in palaeontology. Geol. Today **25**, 134–139. (10.1111/j.1365-2451.2009.00714.x)

[57] Bates KT, Falkingham PL, Breithaupt BH, Hodgetts D, Sellers WI, Manning PL. 2009 How big was ‘Big Al’? Quantifying the effect of soft tissue and osteological unknowns on mass predictions for Allosaurus (Dinosauria: Theropoda). Palaeontol. Electron. **12**, 33. https://doc.rero.ch/record/232381/files/PAL_E1397.pdf

[58] Bates KT *et al*. 2016 Temporal and phylogenetic evolution of the sauropod dinosaur body plan. R. Soc. Open Sci. **3**, 150636. (10.1098/rsos.150636) 27069652 PMC4821263

[59] Bates KT, Falkingham PL, Macaulay S, Brassey C, Maidment SCR. 2015 Downsizing a giant: re-evaluating Dreadnoughtus body mass. Biol. Lett. **11**, 20150215. (10.1098/rsbl.2015.0215) 26063751 PMC4528471

[60] Lacovara KJ *et al*. 2014 A gigantic, exceptionally complete titanosaurian sauropod dinosaur from southern Patagonia, Argentina. Sci. Rep. **4**, 6196. (10.1038/srep06196) 25186586 PMC5385829

[61] Manafzadeh AR. 2023 Joint mobility as a bridge between form and function. J. Exp. Biol. **226**, b245042. (10.1242/jeb.245042) 36700463

[62] Senter P, Robins JH. 2005 Range of motion in the forelimb of the theropod dinosaur Acrocanthosaurus atokensis, and implications for predatory behaviour. J. Zool. **266**, 307–318. (10.1017/s0952836905006989)

[63] White MA, Bell PR, Cook AG, Barnes DG, Tischler TR, Bassam BJ, Elliott DA. 2015 Forearm range of motion in Australovenator wintonensis (Theropoda, Megaraptoridae). PloS One **10**, e0137709. (10.1371/journal.pone.0137709) 26368529 PMC4569425

[64] Mallison H. 2010 CAD assessment of the posture and range of motion of Kentrosaurus aethiopicus Hennig 1915. Swiss J. Geosci. **103**, 211–233. (10.1007/s00015-010-0024-2)

[65] Mallison H. 2010 The digital Plateosaurus II: an assessment of the range of motion of the limbs and vertebral column and of previous reconstructions using a digital skeletal mount. Acta Palaeontol. Pol. **55**, 433–458. (10.4202/app.2009.0075)

[66] Senter P, Sullivan C. 2019 Forelimbs of the theropod dinosaur Dilophosaurus wetherilli: range of motion, influence of paleopathology and soft tissues, and description of a distal carpal bone. Palaeontol. Electron. **22**, 1. (10.26879/900)

[67] Taylor MP, Wedel MJ. 2013 The effect of intervertebral cartilage on neutral posture and range of motion in the necks of sauropod dinosaurs. PloS One **8**, e78214. (10.1371/journal.pone.0078214) 24205163 PMC3812996

[68] Manafzadeh AR, Padian K. 2018 ROM mapping of ligamentous constraints on avian hip mobility: implications for extinct ornithodirans. Proc. R. Soc. B **285**, 20180727. (10.1098/rspb.2018.0727) PMC5998106 29794053

[69] Bonnan MF, Sandrik JL, Nishiwaki T, Wilhite DR, Elsey RM, Vittore C. 2010 Calcified cartilage shape in archosaur long bones reflects overlying joint shape in stress‐bearing elements: implications for nonavian dinosaur locomotion. Anat. Rec. **293**, 2044–2055. (10.1002/ar.21266) 21046673

[70] Brainerd EL, Baier DB, Gatesy SM, Hedrick TL, Metzger KA, Gilbert SL, Crisco JJ. 2010 X‐ray Reconstruction of Moving Morphology (XROMM): precision, accuracy and applications in comparative biomechanics research. J. Exp. Zool. Part Ecol. Genet. Physiol. **313**, 262–279. (10.1002/jez.589) 20095029

[71] Gatesy SM, Baier DB, Jenkins FA, Dial KP. 2010 Scientific rotoscoping: a morphology‐based method of 3‐D motion analysis and visualization. J. Exp. Zool. Part Ecol. Genet. Physiol. **313**, 244–261. (10.1002/jez.588) 20084664

[72] Kambic RE, Roberts TJ, Gatesy SM. 2015 Guineafowl with a twist: asymmetric limb control in steady bipedal locomotion. J. Exp. Biol. **218**, 3836–3844. (10.1242/jeb.126193) 26632457

[73] Kambic RE, Biewener AA, Pierce SE. 2017 Experimental determination of three-dimensional cervical joint mobility in the avian neck. Front. Zool. **14**, 37. (10.1186/s12983-017-0223-z) 28747987 PMC5525307

[74] Manafzadeh AR. 2020 A practical guide to measuring ex vivo joint mobility using XROMM. Integr. Org. Biol. **2**, obaa041. (10.1093/iob/obaa041) 33791578 PMC7810577

[75] Hutson JD, Hutson KN. 2013 Using the American alligator and a repeated-measures design to place constraints on in vivo shoulder joint range of motion in dinosaurs and other fossil archosaurs. J. Exp. Biol. **216**, 275–284. (10.1242/jeb.074229) 22972888

[76] Hutson JD, Hutson KN. 2012 A test of the validity of range of motion studies of fossil archosaur elbow mobility using repeated-measures analysis and the extant phylogenetic bracket. J. Exp. Biol. **215**, 2030–2038. (10.1242/jeb.069567) 22623191

[77] Cobley MJ, Rayfield EJ, Barrett PM. 2013 Inter-vertebral flexibility of the ostrich neck: implications for estimating sauropod neck flexibility. PloS One **8**, e72187. (10.1371/journal.pone.0072187) 23967284 PMC3743800

[78] Dzemski G, Christian A. 2007 Flexibility along the neck of the ostrich (Struthio camelus) and consequences for the reconstruction of dinosaurs with extreme neck length. J. Morphol. **268**, 701–714. (10.1002/jmor.10542) 17514722

[79] Gatesy SM, Manafzadeh AR, Bishop PJ, Turner ML, Kambic RE, Cuff AR, Hutchinson JR. 2022 A proposed standard for quantifying 3‐D hindlimb joint poses in living and extinct archosaurs. J. Anat. **241**, 101–118. (10.1111/joa.13635) 35118654 PMC9178381

[80] Manafzadeh AR, Gatesy SM. 2020 A coordinate-system-independent method for comparing joint rotational mobilities. J. Exp. Biol. **223**, 227108. (10.1242/jeb.227108) 32747453

[81] Bishop PJ, Brocklehurst RJ, Pierce SE. 2023 Intelligent sampling of high‐dimensional joint mobility space for analysis of articular function. Methods Ecol. Evol. **14**, 569–582. (10.1111/2041-210x.14016)

[82] Gatesy SM, Bäker M, Hutchinson JR. 2009 Constraint-based exclusion of limb poses for reconstructing theropod dinosaur locomotion. J. Vertebr. Paleontol. **29**, 535–544. (10.1671/039.029.0213)

[83] Carrano MT. 2001 Implications of limb bone scaling, curvature and eccentricity in mammals and non‐avian dinosaurs. J. Zool. **254**, 41–55. (10.1017/s0952836901000541)

[84] Biewener AA. 1989 Scaling body support in mammals: limb posture and muscle mechanics. Science **245**, 45–48. (10.1126/science.2740914) 2740914

[85] Persons WS, Currie PJ. 2016 An approach to scoring cursorial limb proportions in carnivorous dinosaurs and an attempt to account for allometry. Sci. Rep. **6**, 19828. (10.1038/srep19828) 26813782 PMC4728391

[86] Heinrich RE, Ruff CB, Weishampel DB. 1993 Femoral ontogeny and locomotor biomechanics of Dryosaurus lettowvorbecki (Dinosauria, Iguanodontia). Zool. J. Linn. Soc. **108**, 179–196. (10.1111/j.1096-3642.1993.tb00294.x)

[87] Farlow JO, Smith MB, Robinson JM. 1995 Body mass, bone 'strength indicator,' and cursorial potential of Tyrannosaurus rex. J. Vertebr. Paleontol. **15**, 713–725. (10.1080/02724634.1995.10011257)

[88] Cubo J, Woodward H, Wolff E, Horner JR. 2015 First reported cases of biomechanically adaptive bone modeling in non-avian dinosaurs. PloS One **10**, e0131131. (10.1371/journal.pone.0131131) 26153689 PMC4495995

[89] Broyde S, Dempsey M, Wang L, Cox PG, Fagan M, Bates KT. 2021 Evolutionary biomechanics: hard tissues and soft evidence? Proc. Biol. Sci. **288**, 20202809. (10.1098/rspb.2020.2809) 33593183 PMC7935025

[90] Hirasawa T, Kuratani S. 2018 Evolution of the muscular system in tetrapod limbs. Zool. Lett. **4**, 27. (10.1186/s40851-018-0110-2) PMC6148784 30258652

[91] Romer AS. 1927 The pelvic musculature of ornithischian dinosaurs. Acta Zool. **8**, 225–275. (10.1111/j.1463-6395.1927.tb00653.x)

[92] Witmer LM. 1995 The extant phylogenetic bracket and the importance of reconstructing soft tissues in fossils. Funct. Morphol. Vertebr. Paleontol. **19**, 33.

[93] Gatesy SM. 1990 Caudofemoral musculature and the evolution of theropod locomotion. Paleobiology **16**, 170–186. (10.1017/s0094837300009866)

[94] Bates KT, Benson RBJ, Falkingham PL. 2012 A computational analysis of locomotor anatomy and body mass evolution in Allosauroidea (Dinosauria: Theropoda). Paleobiology **38**, 486–507. (10.1666/10004.1)

[95] Ballell A, Rayfield EJ, Benton MJ. 2022 Walking with early dinosaurs: appendicular myology of the Late Triassic sauropodomorph Thecodontosaurus antiquus. R. Soc. Open Sci. **9**, 211356. (10.1098/rsos.211356) 35116154 PMC8767213

[96] Dilkes DW. 1999 Appendicular myology of the hadrosaurian dinosaur Maiasaura peeblesorum from the Late Cretaceous (Campanian) of Montana. Trans. R. Soc. Edinb. Earth Sci. **90**, 87–125. (10.1017/S0263593300007185)

[97] Maidment SCR, Bates KT, Falkingham PL, VanBuren C, Arbour V, Barrett PM. 2014 Locomotion in ornithischian dinosaurs: an assessment using three‐dimensional computational modelling. Biol. Rev. Camb. Philos. Soc. **89**, 588–617. (10.1111/brv.12071) 24251809

[98] Bates KT, Maidment SC Maidment SCR, Allen V, Barrett PM. 2012 Computational modelling of locomotor muscle moment arms in the basal dinosaur Lesothosaurus diagnosticus: assessing convergence between birds and basal ornithischians. J. Anat. **220**, 212–232. (10.1111/j.1469-7580.2011.01469.x) 22211275 PMC3381616

[99] Allen VR, Kilbourne BM, Hutchinson JR. 2021 The evolution of pelvic limb muscle moment arms in bird-line archosaurs. Sci. Adv. **7**, e2778. (10.1126/sciadv.abe2778) PMC7978429 33741593

[100] Otero A, Allen V, Pol D, Hutchinson JR. 2017 Forelimb muscle and joint actions in Archosauria: insights from Crocodylus johnstoni (Pseudosuchia) and Mussaurus patagonicus (Sauropodomorpha). PeerJ **5**, e3976. (10.7717/peerj.3976) 29188140 PMC5703147

[101] Cerroni MA, Otero A, Novas FE. 2024 Appendicular myology of Skorpiovenator bustingorryi: a first attempt to reconstruct pelvic and hindlimb musculature in an abelisaurid theropod. Anat. Rec. (10.1002/ar.25532) 38989612

[102] Voegele KK, Ullmann PV, Lamanna MC, Lacovara KJ. 2020 Appendicular myological reconstruction of the forelimb of the giant titanosaurian sauropod dinosaur Dreadnoughtus schrani. J. Anat. **237**, 133–154. (10.1111/joa.13176) 32141103 PMC7309294

[103] Brassey CA, Maidment SCR, Barrett PM. 2017 Muscle moment arm analyses applied to vertebrate paleontology: a case study using Stegosaurus stenops Marsh, 1887. J. Vertebr. Paleontol. **37**, e1361432. (10.1080/02724634.2017.1361432)

[104] Persons WS, Currie PJ. 2011 The tail of Tyrannosaurus: reassessing the size and locomotive importance of the M. caudofemoralis in non‐avian theropods. Anat. Rec. **294**, 119–131. (10.1002/ar.21290) 21157923

[105] van Bijlert PA, Schulp AS, AJ ‘Knoek’ AJ. 2021 Natural frequency method: estimating the preferred walking speed of Tyrannosaurus rex based on tail natural frequency. R. Soc. Open Sci. **8**, 201441. (10.1098/rsos.201441) 33996115 PMC8059583

[106] Sellers WI, Pond SB, Brassey CA, Manning PL, Bates KT. 2017 Investigating the running abilities of Tyrannosaurus rex using stress-constrained multibody dynamic analysis. PeerJ **5**, e3420. (10.7717/peerj.3420) 28740745 PMC5518979

[107] Bishop PJ, Cuff AR, Hutchinson JR. 2021 How to build a dinosaur: musculoskeletal modeling and simulation of locomotor biomechanics in extinct animals. Paleobiology **47**, 1–38. (10.1017/pab.2020.46)

[108] Demuth OE, Wiseman ALA, van Beesel J, Mallison H, Hutchinson JR. 2022 Three-dimensional polygonal muscle modelling and line of action estimation in living and extinct taxa. Sci. Rep. **12**, 3358. (10.1038/s41598-022-07074-x) 35233027 PMC8888607

[109] Bates KT, Falkingham PL. 2018 The importance of muscle architecture in biomechanical reconstructions of extinct animals: a case study using Tyrannosaurus rex. J. Anat. **233**, 625–635. (10.1111/joa.12874) 30129185 PMC6183000

[110] Persons, IV, WS, Currie PJ, Norell MA. 2014 Oviraptorosaur tail forms and functions. Acta Palaeontol. Pol. **59**, 553–567. (10.4202/app.2012.0093)

[111] Hutchinson JR. 2004 Biomechanical modeling and sensitivity analysis of bipedal running ability. I. Extant taxa. J. Morphol. **262**, 421–440. (10.1002/jmor.10241) 15352201

[112] Bates KT, Manning PL, Margetts L, Sellers WI. 2010 Sensitivity analysis in evolutionary robotic simulations of bipedal dinosaur running. J. Vertebr. Paleontol. **30**, 458–466. (10.1080/02724630903409329)

[113] Bishop PJ, Falisse A, De Groote F, Hutchinson JR. 2021 Predictive simulations of running gait reveal a critical dynamic role for the tail in bipedal dinosaur locomotion. Sci. Adv. **7**, eabi7348. (10.1126/sciadv.abi7348) 34550734 PMC8457660

[114] Sellers WI, Manning PL, Lyson T, Stevens K, Margetts L. 2009 Virtual palaeontology: gait reconstruction of extinct vertebrates using high performance computing. Palaeontol. Electron. **12**, 26. https://palaeo-electronica.org/2009_3/180/180.pdf

[115] Falkingham PL. 2014 Interpreting ecology and behaviour from the vertebrate fossil track record. J. Zool. **292**, 222–228. (10.1111/jzo.12110)

[116] Gatesy SM, Falkingham PL. 2020 Hitchcock’s leptodactyli, penetrative tracks, and dinosaur footprint diversity. J. Vertebr. Paleontol. **40**. (10.1080/02724634.2020.1781142)

[117] Gatesy SM, Falkingham PL. 2017 Neither bones nor feet: track morphological variation and 'preservation quality'. J. Vertebr. Paleontol. **37**, e1314298. (10.1080/02724634.2017.1314298)

[118] Hitchcock E. 1848 An attempt to discriminate and describe the animals that made the fossil footmarks of the united states, and especially of New England. vol. 3. Cambridge, MA: American Academy of Arts and Science.

[119] Hitchcock E. 1858 Ichnology of New England. A report on the sandstone of the Connecticut valley, especially its fossil footmarks. Boston, MA: William White.

[120] Hitchcock E. 1836 Ornithichnology—description of the foot marks of birds (Ornithichnites) on new red sandstone in Massachusetts. Am. J. Sci. **29**, 307–340.

[121] Alexander RMcN. 1976 Estimates of speeds of dinosaurs. Nature **261**, 129–130. (10.1038/261129a0)

[122] Thulborn T, Wade M. 1984 Dinosaur trackways in the Winton Formation (Mid-Cretaceous) of Queensland, pp. 413–517, vol. 21. Memoirs of the Queensland Museum.

[123] Thulborn RA. Gaits of dinosaurs. In Dinosaur tracks and traces, pp. 257–286. Cambridge, UK: Cambridge University Press. (10.1007/978-94-009-0409-5_9)

[124] Marmol-Guijarro A, Nudds R, Folkow L, Codd J. 2020 Examining the accuracy of trackways for predicting gait selection and speed of locomotion. Front. Zool. **17**, 17. (10.1186/s12983-020-00363-z) 32514280 PMC7254686

[125] Rainforth EC, Manzella M. 2007 Estimating speeds of dinosaurs from trackways: a re-evaluation of assumptions. In Contributions to the Paleontology of New Jersey (II) in Field Guide and Proceedings 41–48, XXIV Ann. Conf. and Field Trip, East Stroudsburg, pp. 12–13. Trenton, NJ: Geological Association of New Jersey.

[126] Henderson D. 2003 Footprints, trackways, and hip heights of bipedal dinosaurs—testing hip height predictions with computer models. Ichnos **10**, 99–114. (10.1080/10420940390257914)

[127] Lallensack JN, Falkingham PL. 2022 A new method to calculate limb phase from trackways reveals gaits of sauropod dinosaurs. Curr. Biol. **32**, 1635–1640.(10.1016/j.cub.2022.02.012) 35240050

[128] Stevens KA, Ernst S, Marty D. 2022 Coupling length: a generalized gleno-acetabular distance measurement for interpreting the size and gait of quadrupedal trackmakers. Swiss J. Geosci. **115**, 18. (10.1186/s00015-022-00418-9)

[129] Goodell Z, Lockley MG, Lucas SG, Schumacher B, Smith J, Trujillo R, Xing L. 2021 A high-altitude sauropod trackway site in the Jurassic of Colorado: the longest known consecutive footprint sequence reveals evidence of strong turning behavior. In Fossil records 7, pp. 101–112, vol. **82**. Albuquerque, NM: New Mexico Museum of Natural History and Science. See https://www.fs.usda.gov/Internet/FSE_DOCUMENTS/fseprd1171502.pdf.

[130] Ishigaki S, Matsumoto Y. 2009 'Off-tracking’-like phenomenon observed in the turning sauropod trackway from the Upper Jurassic of Morocco. Memoi. Fukui. Prefectural. Dinosaur. Museum. **8**, 1–10.

[131] Xing L *et al*. 2015 An unusual sauropod turning trackway from the Early Cretaceous of Shandong Province, China. Palaeogeogr. Palaeoclimatol. Palaeoecol. **437**, 74–84. (10.1016/j.palaeo.2015.07.036)

[132] Xing L *et al*. 2016 Wide-gauge sauropod trackways from the Early Jurassic of Sichuan, China: oldest sauropod trackways from Asia with special emphasis on a specimen showing a narrow turn. Swiss J. Geosci. **109**, 415–428. (10.1007/s00015-016-0229-0)

[133] Lockley MG, Xing L, Kim KS, Meyer CA. 2021 Tortuous trackways: evidence and implications of deviations, turns and changes in direction by dinosaurian trackmakers. Hist. Biol. **33**, 3326–3339. (10.1080/08912963.2020.1865945)

[134] Bishop PJ *et al*. 2017 Using step width to compare locomotor biomechanics between extinct, non-avian theropod dinosaurs and modern obligate bipeds. J. R. Soc. Interface **14**, 20170276. (10.1098/rsif.2017.0276) 28724627 PMC5550975

[135] Wilson JA, Carrano MT. 1999 Titanosaurs and the origin of ‘wide-gauge’ trackways: a biomechanical and systematic perspective on sauropod locomotion. Paleobiology **25**, 252–267. (10.1017/S0094837300026543)

[136] Lockley M, Schulp AS, Meyer CA, Leonardi G, Kerumba Mamani D. 2002 Titanosaurid trackways from the Upper Cretaceous of Bolivia: evidence for large manus, wide-gauge locomotion and gregarious behaviour. Cretac. Res. **23**, 383–400. (10.1006/cres.2002.1006)

[137] Lockley MG, Farlow JO, Meyer C. 1994 Brontopodus and Parabrontopodus ichnogen. nov. and the significance of wide- and narrow-gauge sauropod trackways. Gaia **10**, 135–145.

[138] Romano M, Whyte MA, Jackson SJ. 2007 Trackway ratio: a new look at trackway gauge in the analysis of quadrupedal dinosaur trackways and its implications for ichnotaxonomy. Ichnos **14**, 257–270. (10.1080/10420940601050014)

[139] Castanera D, Pascual C, Canudo JI, Hernández N, Barco JL. 2012 Ethological variations in gauge in sauropod trackways from the Berriasian of Spain. Lethaia **45**, 476–489. (10.1111/j.1502-3931.2012.00304.x)

[140] Farlow JO, Falkingham PL, Therrien F. 2021 Pedal proportions of small and large hadrosaurs and other potentially bipedal ornithischian dinosaurs. Cretac. Res. **127**, 104945. (10.1016/j.cretres.2021.104945)

[141] Castanera D, Vila B, Razzolini NL, Falkingham PL, Canudo JI, Manning PL, Galobart A. 2013 Manus track preservation bias as a key factor for assessing trackmaker identity and quadrupedalism in basal ornithopods. PloS One **8**, e54177. (10.1371/journal.pone.0054177) 23349817 PMC3551957

[142] Butler RJ, Edgar KM, Haller L, Meade LE, Jones HT, Hill O, Scriven S, Reedman C. 2024 Sauropod dinosaur tracks from the Purbeck Group (Early Cretaceous) of Spyway Quarry, Dorset, UK. R. Soc. Open Sci. **11**, 240583. (10.1098/rsos.240583) 39076363 PMC11285821

[143] Falkingham PL, Bates KT, Mannion PD. 2012 Temporal and palaeoenvironmental distribution of manus- and pes-dominated sauropod trackways. J. Geol. Soc. **169**, 365–370. (10.1144/0016-76492011-019)

[144] Farlow JO, Bakker RT, Dattilo BF, Everett Deschner E, Falkingham PL, Harter C, Solis R, Temple D, Ward W. 2020 Thunder lizard handstands: manus-only sauropod trackways from the Glen Rose Formation (Lower Cretaceous, Kendall County, Texas). Ichnos **27**, 167–199. (10.1080/10420940.2019.1698424)

[145] Ishigaki S, Matsumoto Y. 2009 Re-examination of manus-only and manus-dominated sauropod trackways from Morocco. Geol. Q. **53**, 441–448.

[146] Lee YN, Huh M. 2002 Manus-only sauropod tracks in the Uhangri Formation (Upper Cretaceous), Korea and their Paleobiological implications. J. Paleontol. **76**, 558–564. (10.1017/s0022336000037379)

[147] Vila B, Oms O, Galobart À. 2005 Manus‐only titanosaurid trackway from Fumanya (Maastrichtian, Pyrenees): further evidence for an underprint origin. Lethaia **38**, 211–218. (10.1080/00241160510013312)

[148] Bird RT. 1944 Did Brontosaurus ever walk on land? Nat. Hist. **53**, 60–67.

[149] Ishigaki S. 1989 Footprints of swimming sauropods from Morocco. In Dinosaur tracks and traces (eds DD Gillette, MG Lockley), pp. 83–86. Cambridge, UK: Cambridge University Press.

[150] Lockley MG, Rice A. 1990 Did 'Brontosaurus' ever swim out to sea?: Evidence from brontosaur and other dinosaur footprints. Ichnos **1**, 81–90. (10.1080/10420949009386337)

[151] Henderson DM. 2004 Tipsy punters: sauropod dinosaur pneumaticity, buoyancy and aquatic habits. Proc. R. Soc. Lond. B **271**, S180–3. (10.1098/rsbl.2003.0136) PMC1810024 15252977

[152] Falkingham PL, Bates KT, Margetts L, Manning PL. 2011 Simulating sauropod manus-only trackway formation using finite-element analysis. Biol. Lett. **7**, 142–145. (10.1098/rsbl.2010.0403) 20591856 PMC3030862

[153] Milner ARC, Lockley MG, Kirkland JI. 2006 A large collection of well-preserved theropod dinosaur swim tracks from the Lower Jurassic Moenave Formation. In The Triassic–Jurassic terrestrial transition, pp. 44–47. St George, UT: New Mexico Museum of Natural History and Science.

[154] Milner ARC, Lockley MG. 2016 Dinosaur swim track assemblages: characteristics, contexts, and ichnofacies implications. In Dinosaur tracks (eds PL Falkingham, D Marty, A Richter), pp. 153–181. Bloomington, IN: Indiana University Press.

[155] Coombs WP. 1980 Swimming ability of carnivorous dinosaurs. Science **207**, 1198–1200. (10.1126/science.207.4436.1198) 17776854

[156] Ezquerra R, Doublet S, Costeur L, Galton PM, Pérez-Lorente F. 2007 Were non-avian theropod dinosaurs able to swim? Supportive evidence from an Early Cretaceous trackway, Cameros Basin (La Rioja, Spain). Geology **35**, 507. (10.1130/g23452a.1)

[157] Esperante R, Rocha-Rodríguez G, McLarty JA, Biaggi RE, Nick KE, Baltazar HD, Varquera AC. 2023 Diversity of dinosaur tracks and swim traces in a new site in the Upper Cretaceous El Molino Formation, Torotoro National Park, Bolivia. J. South Am. Earth Sci. **128**, 104480. (10.1016/j.jsames.2023.104480)

[158] Xing LD, Lockley MG, Zhang JP, Milner ARC, Klein H, Li DQ, Persons WS, Ebi JF. 2013 A new early cretaceous dinosaur track assemblage and the first definite non-avian theropod swim trackway from China. Chin. Sci. Bull. **58**, 2370–2378. (10.1007/s11434-013-5802-6)

[159] Romano M, Whyte MA. 2015 Could stegosaurs swim? Suggestive evidence from the Middle Jurassic tracksite of the Cleveland Basin, Yorkshire, UK. Proc. Yorks. Geol. Soc. **60**, 227–233. (10.1144/pygs2015-354)

[160] Falkingham PL, Turner ML, Gatesy SM. 2020 Constructing and testing hypotheses of dinosaur foot motions from fossil tracks using digitization and simulation. Palaeontology **63**, 865–880. (10.1111/pala.12502)

[161] Gatesy SM. 2001 Skin impressions of Triassic theropods as records of foot movement. Bull. Mus. Comp. Zool. **156**, 137–149.

[162] Gatesy SM, Middleton KM, Jr FAJ, Shubin NH. 1999 Three-dimensional preservation of foot movements in Triassic theropod dinosaurs. Nature **399**, 141–144. (10.1038/20167)

[163] Avanzini M, Piñuela L, García-Ramos JC. 2012 Late Jurassic footprints reveal walking kinematics of theropod dinosaurs. Lethaia **45**, 238–252. (10.1111/j.1502-3931.2011.00276.x)

[164] Cobos A, Gasco F, Royo-Torres R, Lockley MG, Alcala L. 2016 Dinosaur tracks as ‘four-dimensional phenomena’ reveal how different species moved. In Dinosaur tracks the next steps (eds PL Falkingham, D Marty, A Richter), pp. 245–255. Bloomington, IN: Indiana University Press.

[165] Allen JRL. 1997 Subfossil mammalian tracks (Flandrian) in the Severn Estuary, S. W. Britain: mechanics of formation, preservation and distribution. Phil. Trans. R. Soc. B **352**, 481–518. (10.1098/rstb.1997.0035)

[166] Manning PL. 2004 A new approach to the analysis and interpretation of tracks: examples from the dinosauria. Geol. Soc. Lond. Spec. Publ. **228**, 93–123. (10.1144/gsl.sp.2004.228.01.06)

[167] Milàn J, Clemmensen LB, Bonde N. 2004 Vertical sections through dinosaur tracks (Late Triassic lake deposits, East Greenland)—undertracks and other subsurface deformation structures revealed. Lethaia **37**, 285–296. (10.1080/00241160410002036)

[168] Turner ML, Falkingham PL, Gatesy SM. 2020 It’s in the loop: shared sub-surface foot kinematics in birds and other dinosaurs shed light on a new dimension of fossil track diversity. Biol. Lett. **16**, 20200309. (10.1098/rsbl.2020.0309) 32603644 PMC7423045

[169] Novotny J, Tveite J, Turner ML, Gatesy S, Drury F, Falkingham P, Laidlaw DH. 2019 Developing virtual reality visualizations for unsteady flow analysis of dinosaur track formation using scientific sketching. IEEE Trans. Vis. Comput. Graph. **25**, 2145–2154. (10.1109/tvcg.2019.2898796) 30908229

[170] Milàn J, Christiansen P, Mateus O. 2005 A three-dimensionally preserved sauropod manus impression from the Upper Jurassic of Portugal: implications for sauropod manus shape and locomotor mechanics. Kaupia **14**, 47–52.

[171] Farlow JO *et al*. 2012 Dinosaur tracksites of the Paluxy River Valley (Glen Rose Formation, Lower Cretaceous), Dinosaur Valley State Park, Somervell County, Texas. Proc. V Int. Symp. Dinosaur Palaeontol. Environ. 41–69.

[172] Milàn J, Bromley RG. 2008 The impact of sediment consistency on track and undertrack morphology: experiments with emu tracks in layered cement. Ichnos **15**, 19–27. (10.1080/10420940600864712)

[173] Thulborn RA. 1990 Dinosaur tracks. London, UK: Chapman & Hall.

[174] Farlow JO. 1981 Estimates of dinosaur speeds from a new trackway site in Texas. Nature **294**, 747–748. (10.1038/294747a0)

[175] Thulborn RA, Wade M. 1979 Dinosaur stampede in the Cretaceous of Queensland. Lethaia **12**, 275–279. (10.1111/j.1502-3931.1979.tb01008.x)

[176] Navarro-Lorbés P, Ruiz J, Díaz-Martínez I, Isasmendi E, Sáez-Benito P, Viera L, Pereda-Suberbiola X, Torices A. 2021 Fast-running theropods tracks from the Early Cretaceous of La Rioja, Spain. Sci. Rep. **11**, 23095. (10.1038/s41598-021-02557-9) 34887437 PMC8660891

